# RPP40 is a prognostic biomarker and correlated with tumor microenvironment in uterine corpus endometrial carcinoma

**DOI:** 10.3389/fonc.2022.957472

**Published:** 2022-08-24

**Authors:** Jianming Tang, Xiaoli Tian, Jie Min, Ming Hu, Li Hong

**Affiliations:** ^1^ Department of Obstetrics and Gynecology, Renmin Hospital of Wuhan University, Wuhan, China; ^2^ Department of Pathology, Renmin Hospital of Wuhan University, Wuhan, China

**Keywords:** RPP40, uterine corpus endometrial carcinoma, immune infiltration, extracellular matrix, tumor microenvironment, prognosis

## Abstract

Ribonuclease P/MRP Subunit P40 (RPP40), a component of ribonuclease P and multimeric ribonuclease P complex, was reported as one of the promoting factors for the chemoresistance of acute myeloid leukemia and a recurrence predictor of early-stage triple-negative breast cancer. However, the functional role of RPP40 in uterine corpus endometrial carcinoma (UCEC) is unclear. In this study, comprehensive bioinformatic analyses were conducted to explore the predictive role of RPP40 on UCEC diagnosis and prognosis, as well as the underlying mechanism. Differential analyses of multiple databases showed that both messenger RNA (mRNA) and the protein expression of RPP40 were significantly upregulated in UCEC tumor tissues. Furthermore, the RPP40 mRNA expression level was significantly correlated with the clinicopathological characteristics of UCEC patients, including the clinical stage, primary therapy outcome, histological type, histologic grade, overall survival event, disease-specific survival event, and progression-free interval event. Receiver operating characteristic (ROC) analysis showed that RPP40 was a reliable predictor for UCEC diagnosis with an area under the curve (AUC) of 0.775, a sensitivity of 0.829, and a specificity of 0.719. Kaplan–Meier, Cox regression, and nomogram analyses showed that high RPP40 expression was an independent prognostic factor for the 1-year, 3-year, and 5-year survival of UCEC patients. In addition, the enrichment analysis of RPP40-associated differentially expressed genes and correlation analyses showed that the expression of RPP40 was correlated with the regulation of extracellular matrix and immune cell infiltration. In conclusion, the upregulation of RPP40 is significantly correlated with the poor survival and tumor microenvironment of UCEC, suggesting that RPP40 is a promising biomarker of poor prognosis and a potential target of chemotherapy or immunotherapy in UCEC.

## Introduction

Uterine corpus endometrial carcinoma (UCEC) is the third most commonly diagnosed gynecological cancer and the seventh most common malignant tumor in women worldwide (1, 2). Over 60,000 new cases are expected next year in American women (3). Generally, early screening and therapies can significantly reduce the incidence, recurrence, and mortality of UCEC. Nevertheless, the patients in advanced stages usually respond poorly to conventional treatments, with a 5-year survival rate as low as 17% (4). In recent years, evolving medical drugs and technologies have slowed the decline in the long-term survival rate in UCEC patients. However, novel prognostic biomarkers and therapeutic targets for improving the survival rate of UCEC patients still need continuous exploration.

The tumor microenvironment (TME), composed of multiple cellular and molecular components, has been implicated in cancer cell survival, proliferation, invasion, and therapeutic response (5–7). Various members of TME, such as cancer-associated fibroblasts (CAFs), immune cells, extracellular matrix (ECM), cytokines, and chemokines, act together to regulate phenotypes, antitumor immunity, and the therapeutic response of malignant tumors (5–8). The metabolic and biologic changes of malignant cells driven by oncogenes can influence the TME to suppress antineoplastic immune responses and induce therapeutic resistance (7). Meanwhile, this also reveals a novel strategy for cancer therapy to remodel the TME by targeting hub oncogenes and related signaling pathways.

Ribonuclease P/MRP Subunit P40 (RPP40), a 40-KDa protein subunit of ribonuclease P (RNase P), was reported to enable RNase P RNA binding activity and then contribute to the generation of mature tRNA molecules (9–11). Moreover, RPP40 is also a component of the multimeric ribonuclease P (MRP) complex, which cleaves pre-rRNA sequences (12). At present, the molecular function of RPP40 remains unclear since there are few studies concerning it. As other components of RNase P or MRP, both RPP25 and RPP30 were reported as reliable prognostic risk factors for glioblastoma multiforme (11, 13) and also have been reported to promote the proliferation, migration, invasion, and cell cycle program of cervical cancer cells (14). Similarly, RPP40 was also regarded as one of the promoting factors for the chemoresistance of acute myeloid leukemia (15) and recurrence predictor of early-stage triple-negative breast cancer (16). Furthermore, the result of bioinformatics analysis in this study showed that RPP40 was one of the potential prognostic genes for UCEC (**Supplementary File 1**). Therefore, we speculated that RPP40 might be a potential prognostic biomarker or therapeutic target of UCEC and might be involved in its tumorigenesis or progression.

Based on the above speculation, we first analyzed the expression difference, survival prognosis, and possible molecular function of RPP40 in UCEC in this study. We found that both mRNA and protein expression were significantly upregulated in UCEC tumor tissues. Moreover, RPP40 was an effective diagnostic and prognostic predictor of UCEC. In addition, gene enrichment analysis revealed that RPP40 was involved in regulating the TME, especially ECM dysregulation and immune cell infiltration.

## Materials and methods

### TCGA database and data processing

Transcriptional expression data of 21 types of cancer and paired clinical data of UCEC were downloaded from the The Cancer Genome Atlas (TCGA) database (https://portal.gdc.cancer.gov/). RNA sequencing data were transformed from the format of fragments per kilobase per million (FPKM) to transcripts per million reads (TPM) for further analyses. This study did not require ethical approval since the research data we used was acquired from public online databases. Then, the mRNA expression differences between tumor tissues and normal tissues were determined in 21 types of cancer, including bladder urothelial carcinoma (BLCA), breast-invasive carcinoma (BRCA), cervical squamous cell carcinoma and endocervical adenocarcinoma (CESC), cholangiocarcinoma (CHOL), colon adenocarcinoma (COAD), esophageal carcinoma (ESCA), glioblastoma multiforme (GBM), head and neck squamous cell carcinoma (HNSC), kidney chromophobe (KICH), kidney renal clear cell carcinoma (KIRC), kidney renal papillary cell carcinoma (KIRP), liver hepatocellular carcinoma (LIHC), lung adenocarcinoma (LUAD), lung squamous cell carcinoma (LUSC), pancreatic adenocarcinoma (PAAD), pheochromocytoma and paraganglioma (PCPG), prostate adenocarcinoma (PRAD), rectum adenocarcinoma (READ), stomach adenocarcinoma (STAD), thyroid carcinoma (THCA), and UCEC.

### The University of Alabama at Birmingham cancer data analysis portal and clinical proteomic tumor analysis consortium

The University of Alabama at Birmingham Cancer data analysis Portal (UALCAN; http://ualcan.path.uab.edu/analysis-prot.html) is a public online database that provides protein expression analysis option using data from Clinical Proteomic Tumor Analysis Consortium (CPTAC) and the International Cancer Proteogenome Consortium (ICPC) datasets (17, 18). In this study, we compared the protein expression difference between primary UCEC tumor samples (n=100) and normal endometrial samples (n=31) using the data from CPTAC on ULCAN. The z-value represents standard deviations from the median across samples for UCEC. Log2 spectral count ratio values from CPTAC were first normalized within each sample profile and then normalized across samples.

### The Human Protein Atlas

The Human Protein Atlas (HPA; https://www.proteinatlas.org/) is a public database that contains the protein expression data of human protein-coding genes (19, 20). The immunohistochemical staining pictures of normal and tumor tissues were publicly available in this database. In this study, we compared the protein expression of RPP40 between UCEC tumor tissue and normal endometrial tissue on HPA.

### Study design, grouping, and sample size

**Figure 1:**
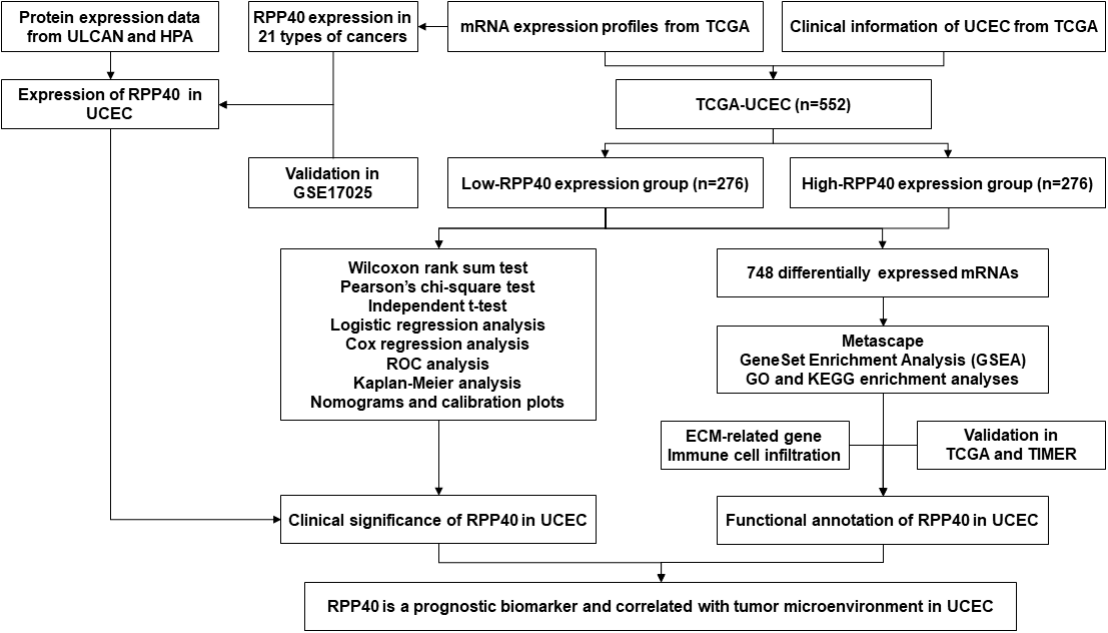
Flow diagram of this study.

The study flowchart is illustrated in **Figure 1**. In this study, 552 UCEC patients were divided into two groups, high- and low-RPP40-expression groups, according to the median value of RPP40 expression in UCEC tumor samples. There were 276 patients in each group. Then the patients were divided into subgroups for further analyses based on each clinicopathological characteristics. The sample size of each subgroup is shown in the **Supplementary Table 1**.

### Correlation analysis for RPP40 expression and clinicopathological characteristics of uterine corpus endometrial carcinoma patients

The clinicopathological characteristics of UCEC patients between high- and low-RPP40-expression groups were compared using the Wilcoxon rank sum test (continuous variables) or Pearson’s chi-square test (rank variables). Secondly, the correlation research of RPP40 expression with clinicopathological characteristics was investigated *via* logistic analysis. Next, the expression differences of RPP40 among different subgroups of clinicopathological characteristics were compared by an independent t-test. A *p*-value <0.05 was regarded statistically significant.

### Clinical significance evaluation of RPP40 expression in uterine corpus endometrial carcinoma

To evaluate the predictive value of RPP40 in UCEC diagnosis, receiver operating characteristic (ROC) analysis was conducted using an R package of “pROC” (21). Next, Kaplan–Meier (K-M), univariate, and multivariate Cox regression analyses were employed for prognosis analysis, including overall survival (OS), disease-specific survival (DSS), and progression-free interval (PFI). The R packages “rms” and “survival” were applied to construct nomograms and calibration plots. The R packages “forestplot” and “survival” were used for the clinicopathological subgroup study. All survival data in this study were acquired from the published research (22). All the above analyses were all accomplished with R (v3.6.3), and a *p*-value <0.05 was considered as the statistical threshold.

### RPP40-related differentially expressed genes in uterine corpus endometrial carcinoma tumors

Differentially expressed genes (DEGs) between high- and low-RPP40-expression groups were screened out using R package “DESeq2” (23). Furthermore, The R package “ggplot2” was used to illustrate results as volcano plots and heatmaps. *P*<0.05 and |log2 Fold change|>1.0 were set as thresholds for DEGs with statistical significance.

### Enrichment analysis of RPP40-associated DEGs in uterine corpus endometrial carcinoma tumors

The DEGs with significance were then processed for enrichment analysis on the Metascape database (http://metascape.org) (24), with the analysis thresholds of counts≥3, enrichment factors>1.5, and *P*-value<0.01. Furthermore, the R package “clusterProfiler” (25) was utilized for the gene set enrichment analysis (GSEA) (26) of the DEGs between two groups, as well as the Gene ontology (GO) and Kyoto Encyclopedia of Genes and Genomes (KEGG) enrichment analyses. The data set of “c2.cp.v7.2.symbols.gmt” from MSigDB collections were selected as reference gene sets in GSEA analysis. The number of analysis permutations was set to 1000, and False discovery rate (FDR)<0.25 and adjusted *P*-value<0.05 were set as analysis thresholds in GSEA.

### Association of RPP40 and immune cell infiltration in uterine corpus endometrial carcinoma tumors

Firstly, a single-sample GSEA method from R package “GSVA” (27) was used to analyze the correlation between the RPP40 expression and infiltration of 24 common immune cell types (28), including dendritic cells (DCs), activated DCs (aDCs), B cells, CD8 T cells, cytotoxic cells, eosinophils, immature DCs (iDCs), macrophages, mast cells, neutrophils, natural killer (NK) cells, NK CD56bright cells, NK CD56dim cells, plasmacytoid DCs (pDCs), T cells, T helper cells, T central memory (Tcm), T effector memory (Tem), T follicular helper (TFH), T gamma delta (Tgd), Th1 cells, Th17 cells, Th2 cells, and Treg. Secondly, the immune cell infiltration levels between high- and low-RPP40-expression groups were compared by an independent-samples t-test. Furthermore, the association between the RPP40 expression and gene marker levels of immune cells in UCEC tumor tissues was determined *via* the Tumor IMmune Estimation Resource (TIMER) database (https://cistrome.shinyapps.io/timer/). A *p*-value <0.05 was regarded statistically significant in all above analyses.

## Results

### Expression profiles of RPP40 in pan-cancer perspective

**Figure 2:**
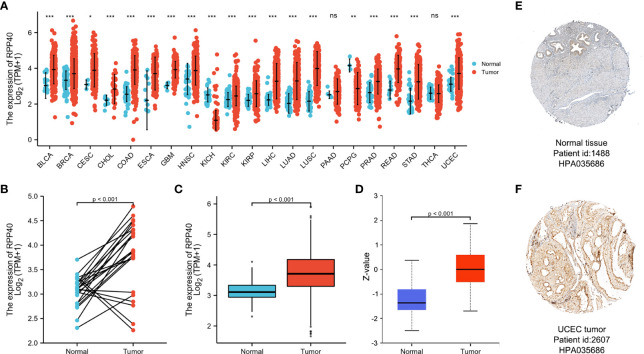
Expression of Ribonuclease P/MRP Subunit P40 (RPP40) in pan-cancer perspective. **(A)** The comparison of RPP40 mRNA expression between tumor and normal tissues in different cancer types based on the TCGA database. **(B)** Paired analysis of the mRNA expression levels of RPP40 in 23 UCEC samples and matched normal samples from the TCGA database. **(C)** Unpaired analysis of the mRNA expression levels of RPP40 in 552 UCEC samples and 35 normal samples from the TCGA database. **(D)** The protein expression difference of RPP40 between UCEC tumor tissues and normal endometrial tissues based on CPTAC. **(E, F)** The immunohistochemical staining of RPP40 protein in normal endometrial sample **(E)** and UCEC tumor sample **(F)** based on Human Protein Atlas. ns, *P*≥0.05; *P < 0.05; **P < 0.01; ***P < 0.001.

To determine the mRNA expression pattern of RPP40 in different cancers, the mRNA expression data of RPP40 in the tumors and corresponding normal tissues of different cancer types based on the TCGA database were analyzed. As shown in **Figure 2A**, when compared with normal samples, the RPP40 mRNA expression of tumor samples were significantly upregulated in the tumor samples of 17 cancer types, including BLCA, BRCA, CESC, CHOL, COAD, ESCA, GBM, HNSC, KIRC, KIRP, LIHC, LUAD, LUSC, PRAD, READ, STAD, and UCEC according to the TCGA database. These results indicate that the mRNA expression of RPP40 is significantly upregulated in a variety of cancer types.

Next, the mRNA expression characteristic of RPP40 in UCEC was further determined. As shown in **Figure 2B**, paired data analysis showed that the mRNA expression levels of RPP40 in UCEC tumor tissues were significantly upregulated than those in normal endometrial tissues (n=23) according to the TCGA database. Similarly, as shown in **Figure 2C**, the RPP40 mRNA expression levels of UCEC tumor tissues (n=552) were significantly increased than those of normal tissues (n=35) in unpaired data analysis. We also validated the mRNA expression of RPP40 in the GSE17025 dataset. As shown in **Supplementary Figure 1**, RPP40 mRNA expression levels in UCEC tumor tissues (n=91) were significantly upregulated than those in normal tissues (n=12). Furthermore, the protein expression of RPP40 in UCEC was analyzed on both UALCAN and HPA databases. As shown in **Figure 2D**, the protein expression of RPP40 in primary UCEC (CPTAC samples, n=100) was significantly higher than those in normal endometrial tissues (CPTAC samples, n=31). As same as the research result from CPTAC samples, immunohistochemical staining results from the HPA database also confirmed that the protein level of RPP40 was markedly upregulated in UCEC tumor tissues (**Figures 2E, F**). These results indicate that both the mRNA and protein expression of RPP40 are significantly upregulated in UCEC tumor tissues.

### Association between RPP40 expression and clinicopathological characteristics in uterine corpus endometrial carcinoma patients

**Figure 3:**
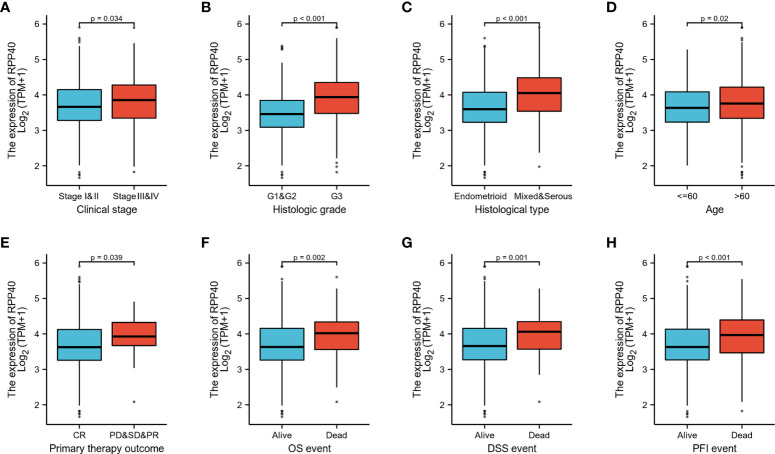
RPP40 expression is associated with clinicopathological characteristics in UCEC patients. The expression differences of RPP40 between distinct subgroups of UCEC patients are based on different clinicopathological characteristics, including clinical stage **(A)**, histological grade **(B)**, histological type **(C)**, age **(D)**, primary therapy outcome **(E)**, OS event **(F)**, DSS event **(G)**, and PFI event **(H)**.

To evaluate the potential clinical significance of RPP40 in UCEC, 552 UCEC patients were divided into two groups, high- (n=276) and low- (n=276) RPP40-expression groups, based on the RPP40 mRNA expression levels in UCEC tumor tissues. Then, the clinicopathological characteristics of UCEC patients between different RPP40 expression levels were compared (**Table 1**). The results showed that the RPP40 mRNA expression level was significantly correlated with the clinical stage, primary therapy outcome, histological type, histologic grade, OS event, DSS event, and PFI event of UCEC patients. Moreover, logistics analysis was applied to further confirm the correlation between RPP40 expression and clinicopathological characteristics. As shown in **Table 2**, RPP40 expression was positively correlated with the clinical stage (OR=1.617, *P*=0.011), histological grade (OR=3.280, *P*<0.001), histological type (OR=3.166, *P*<0.001), and primary therapy outcome (OR=2.864, *P*=0.004). Moreover, we also investigated the expression differences of RPP40 among different subgroups of clinicopathological characteristics. The result showed that RPP40 expression was significantly increased in patients with clinical stages III–IV (**Figure 3A**), histological grade G3 (**Figure 3B**), the histological type of serous and mixed (**Figure 3C**), age over 60 years old (**Figure 3D**), the primary therapy outcome of PD&SD&PR (**Figure 3E**), and dead patients in the survival event of OS, DSS, and PFI (**Figures 3F–H**). At the same time, there were no significant differences in RPP40 expression between the two subgroups of BMI, residual tumor, tumor invasion, menopause status, hormone therapy, diabetes, radiation therapy, and surgical approach (**Supplementary Figure 2**).

**Table 1:** Clinicopathological characteristics of uterine corpus endometrial carcinoma (UCEC) patients with differential RPP40 expression.

Characteristic	Low-RPP40 expression (N = 276)	High-RPP40 expression (N = 276)	*P*-value
Clinical stage, n (%)			0.038
Stage I	185 (33.5%)	157 (28.4%)	
Stage II	25 (4.5%)	26 (4.7%)	
Stage III	57 (10.3%)	73 (13.2%)	
Stage IV	9 (1.6%)	20 (3.6%)	
Primary therapy outcome, n (%)			< 0.001
PD	8 (1.7%)	12 (2.5%)	
SD	3 (0.6%)	3 (0.6%)	
PR	0 (0%)	12 (2.5%)	
CR	238 (49.6%)	204 (42.5%)	
Race, n (%)			0.176
Asian	11 (2.2%)	9 (1.8%)	
Black or African American	45 (8.9%)	63 (12.4%)	
White	195 (38.5%)	184 (36.3%)	
Age, n (%)			0.088
<=60	113 (20.6%)	93 (16.9%)	
>60	161 (29.3%)	182 (33.2%)	
BMI, n (%)			0.179
<=30	99 (19.1%)	113 (21.8%)	
>30	163 (31.4%)	144 (27.7%)	
Histological type, n (%)			< 0.001
Endometrioid	234 (42.4%)	176 (31.9%)	
Mixed	11 (2%)	13 (2.4%)	
Serous	31 (5.6%)	87 (15.8%)	
Residual tumor, n (%)			0.857
R0	193 (46.7%)	182 (44.1%)	
R1	10 (2.4%)	12 (2.9%)	
R2	8 (1.9%)	8 (1.9%)	
Histologic grade, n (%)			< 0.001
G1	74 (13.7%)	24 (4.4%)	
G2	73 (13.5%)	47 (8.7%)	
G3	125 (23.1%)	198 (36.6%)	
Tumor invasion (%), n (%)			0.697
<50	134 (28.3%)	125 (26.4%)	
>=50	116 (24.5%)	99 (20.9%)	
Menopause status, n (%)			0.500
Pre	20 (4%)	15 (3%)	
Peri	10 (2%)	7 (1.4%)	
Post	223 (44.1%)	231 (45.7%)	
Hormones therapy, n (%)			0.416
No	148 (43%)	149 (43.3%)	
Yes	27 (7.8%)	20 (5.8%)	
Diabetes, n (%)			0.403
No	171 (37.9%)	157 (34.8%)	
Yes	58 (12.9%)	65 (14.4%)	
Radiation therapy, n (%)			0.980
No	142 (26.9%)	137 (26%)	
Yes	125 (23.7%)	123 (23.3%)	
Surgical approach, n (%)			0.874
Minimally invasive	103 (19.4%)	105 (19.8%)	
Open	163 (30.8%)	159 (30%)	
OS event, n (%)			< 0.001
Alive	246 (44.6%)	212 (38.4%)	
Dead	30 (5.4%)	64 (11.6%)	
DSS event, n (%)			0.003
Alive	256 (46.5%)	231 (42%)	
Dead	20 (3.6%)	43 (7.8%)	
PFI event, n (%)			0.005
Alive	226 (40.9%)	197 (35.7%)	
Dead	50 (9.1%)	79 (14.3%)	

**Table 2:** Logistic regression analysis of association between clinicopathological characteristics and RPP40 expression in UCEC patients.

Characteristics	Odds Ratio (95%CI)	*P*-value
Clinical stage (Stage III–IV vs. Stage I–II)	1.617 (1.116-2.353)	0.011
Histologic grade (G3 vs. G1–2)	3.280 (2.293-4.724)	<0.001
Histological type (Mixed and Serous vs. Endometrioid)	3.166 (2.114-4.808)	<0.001
Age (>60 vs. ≤60)	1.374 (0.972-1.945)	0.073
BMI (>30 vs. ≤30)	0.774 (0.544-1.099)	0.152
Menopause status (Post vs. Pre and Peri)	1.413 (0.794-2.549)	0.243
Primary therapy outcome (PD and SD and PR vs. CR)	2.864 (1.422-6.156)	0.004
Residual tumor (R1 and R2 vs. R0)	1.178 (0.603-2.317)	0.630
Tumor invasion (%) (≥50 vs. <50)	0.915 (0.636-1.314)	0.630
Hormones therapy (Yes vs. No)	0.736 (0.391-1.364)	0.333
Diabetes (Yes vs. No)	1.221 (0.806-1.851)	0.346
Radiation therapy (Yes vs. No)	1.020 (0.724-1.436)	0.910
Surgical approach (Open vs. Minimally Invasive)	0.957 (0.675-1.356)	0.804

### Predictive values of RPP40 for the diagnosis and prognosis of UCEC patients

**Figure 4:**
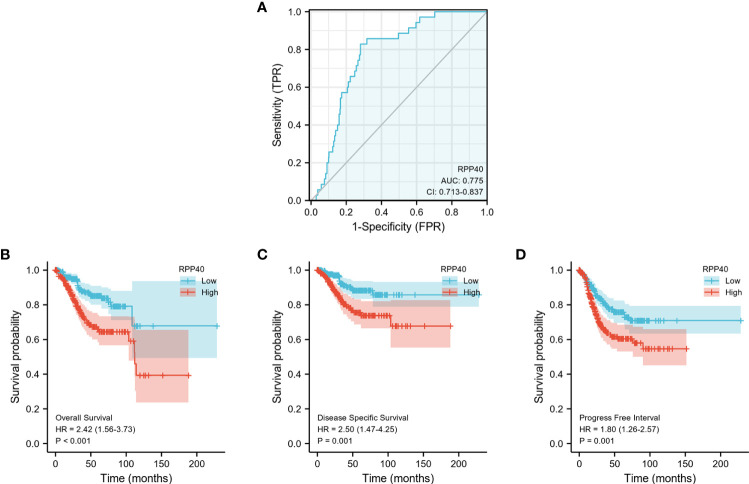
Predictive value of RPP40 expression for diagnosis and survival in UCEC patients. **(A)** ROC curve analysis was used to evaluate the performance of RPP40 for UCEC diagnosis. **(B-D)** K-M analyses were used to compare OS **(B)**, DSS **(C)**, and PFI **(D)** between high- and low-RPP40-expression groups of UCEC patients.

ROC curve analysis was conducted to further explore the clinical significance of RPP40 in UCEC patients. The result showed that RPP40 was a reliable predictive biomarker for the diagnosis of UCEC, with an area under the curve (AUC) of 0.775, a sensitivity of 0.829, and a specificity of 0.719 (**Figure 4A**). Furthermore, K-M analyses were conducted to evaluate the prognostic value of RPP40 in UCEC patients. As shown in **Figures 4B–D**, the OS (HR=2.42, *P*<0.001), DSS (HR=2.50, *P*=0.001), and PFI (HR=1.80, *P*=0.001) of the patients in high-RPP40-expression patients were all significantly shorter than those in low-RPP40-expression patients. Moreover, to further evaluate the prognostic value of RPP40 in UCEC patients, univariate and multivariate Cox regression analyses were accomplished in this study. As shown in **Table 3**, RPP40 expression was an independent risk factor for OS (HR=2.491, *P*=0.007), DSS (HR= 3.060, *P*=0.011) and PFI (HR=1.811, *P*=0.045) in multivariate Cox regression. Moreover, the clinical stage and primary therapy outcome also showed prognostic values for OS, DSS, and PFI, the residual tumor also showed a prognostic value for DSS, and the histological type also showed a prognostic value for PFI in multivariate Cox regression analyses.

**Table 3:** Cox regression analysis for clinical outcomes in UCEC patients.

Characteristics	HR (95% CI) for OS		HR (95% CI) for DSS		HR (95% CI) for PFI	
	Univariate	Multivariate	Univariate	Multivariate	Univariate	Multivariate
Clinical stage (III–IV vs. I–II)	3.543***	3.849***	7.030***	5.641**	3.169***	2.692**
Histologic grade (G3 vs. G1–2)	3.281***	1.062^NS^	7.851***	1.615^NS^	2.088***	0.673^NS^
Histological type (Mixed and Serous vs. Endo)	2.628***	1.286^NS^	3.572***	1.450^NS^	2.109***	2.079*
Age (>60 vs. ≤60)	1.847*	1.549^NS^	0.215^NS^		1.353^NS^	
BMI (>30 vs. ≤30)	0.967^NS^		0.948^NS^		1.046^NS^	
Menopause status (Post vs. Pre and Peri)	1.050^NS^		1.214^NS^		1.637^NS^	
Residual tumor (R1 and R2 vs. R0)	3.101***	2.201^NS^	5.310***	3.309*	2.724***	1.963^NS^
Diabetes (Yes vs. No)	1.172^NS^		1.195^NS^		1.169^NS^	
Surgical approach (Open vs. Minimally Invasive)	0.709^NS^		0.661^NS^		0.629*	0.587^NS^
Primary therapy outcome (PD and SD and PR vs. CR)	7.729***	3.409**	13.602***	5.412***	8.331***	6.283***
Tumor invasion (%) (≥50 vs. <50)	2.813***	0.259^NS^	3.281***	1.026^NS^	1.885**	1.439NS
RPP40 (High vs. Low)	2.417***	2.491**	2.497***	3.060*	1.799**	1.811*

^NS^P>0.05, *P<0.05, **P<0.01, ***P<0.001.

**Figure 5:**
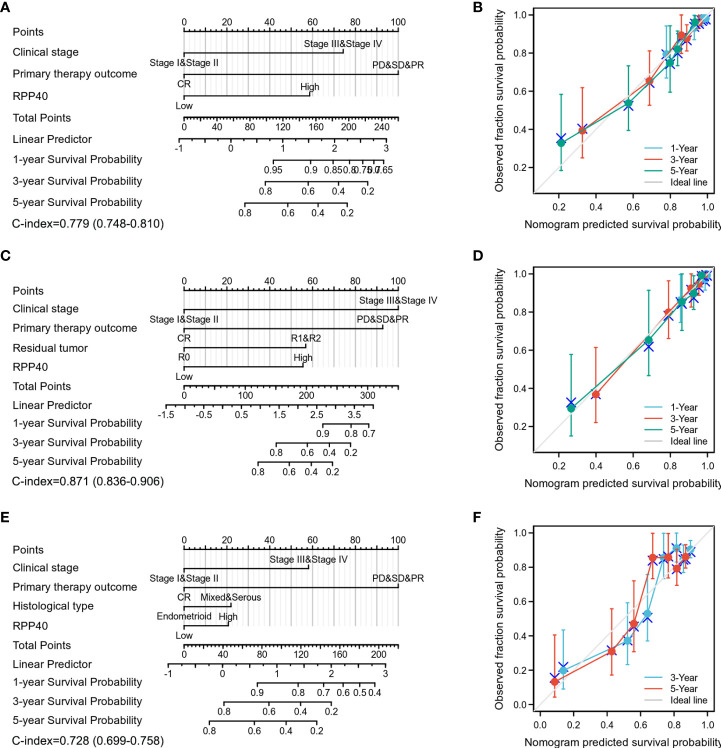
Construction and validation of nomograms in UCEC prognosis based on RPP40 expression. The nomograms were constructed to establish RPP40 expression-based risk scoring models for 1-, 3-, and 5-year OS **(A)**, DSS **(C)**, and PFI **(E)**. Calibration plots were drawn to validate the efficiency of nomograms for OS **(B)**, DSS **(D)**, and PFI **(F)**.

Next, all the significant prognostic factors in multivariate Cox regression analyses were used for prognostic nomogram construction. Then, the corresponding calibration curves were drawn for further testing the efficiency of each nomogram. As shown in **Figure 5**, the clinical stage, primary therapy outcome, and RPP40 expression were used to predict 1-, 3-, and 5-year OS with a C-index of 0.779 (**Figures 5A, B**). The clinical stage, primary therapy outcome, residual tumor, and RPP40 expression were used to predict 1-, 3-, and 5-year DSS with a C-index of 0.871 (**Figures 5C, D**). The clinical stage, primary therapy outcome, histological type, and RPP40 expression were used to predict 1-, 3-, and 5-year PFI with a C-index of 0.728 (**Figures 5E, F**). The calibration curves showed a desirable prediction of OS and DSS nomograms for the 1-, 3-, and 5-year clinical outcomes, with a slightly overestimated mortality in patients with predicted mortality higher than 50% in the 3- and 5-year prediction of OS. These results indicated that RPP40 was a reliable prognostic biomarker for UCEC, especially in predicting OS and DSS.

### Prognostic values of RPP40 in uterine corpus endometrial carcinoma clinicopathological subgroups

**Figure 6:**
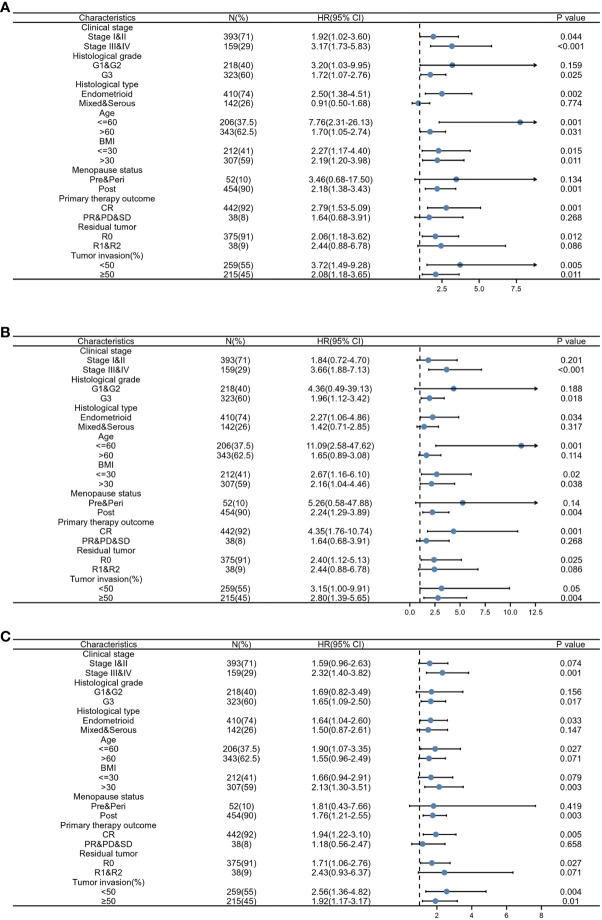
Prognostic performance of RPP40 on clinical outcomes in different subgroups of UCEC patients. Patients were divided into different subgroups according to clinical stage, histological grade, histological type, age, BMI, menopause status, primary therapy outcome, residual tumor, and tumor invasion. For each subgroup, the prognostic performance of RPP40 on OS **(A)**, DSS **(B)**, and PFI **(C)** were evaluated by Cox regression, and the results are presented as a hazard ratio. The bar represents the 95% confidence interval of the hazard ratio.

To further confirm the prognostic value of RPP40 in UCEC, a subgroup study of each clinicopathological factor was conducted by Cox regression analysis, and the results of subgroup analyses were presented as forest plots. As shown in **Figure 6A**, the upregulation of RPP40 was a risk factor for OS in UCEC patients with clinical stage I–II (HR=1.920, *P*=0.044), clinical stage III–IV (HR=3.170, *P*<0.001), histological grade G3 (HR=1.720, *P*=0.025), histological type of endometrioid (HR=2.500, *P*=0.002), age below 60 years old (HR=7.760, *P*=0.001), age over 60 years old (HR=1.700, *P*=0.031), a BMI less than 30 kg/m^2^ (HR=2.270, *P*=0.015), a BMI over 30 kg/m^2^ (HR=2.190, *P*=0.011), postmenopause status (HR=2.180, *P*=0.001), the primary therapy outcome of CR (HR=2.790, *P*=0.001), residual tumor R0 (HR=2.060, *P*=0.012), tumor invasion less than 50% of the muscular layer (HR=3.720, *P*=0.005), or tumor invasion over 50% of the muscular layer (HR=2.080, *P*=0.011). Furthermore, as shown in **Figure 6B**, the upregulation of RPP40 was a risk factor for DSS in patients with clinical stage III–IV (HR=3.660, *P*<0.001), histological grade G3 (HR=1.960, *P*=0.018), a histological type of endometrioid (HR=2.270, *P*=0.034), age below 60 years old (HR=11.090, *P*=0.001), BMI less than 30 kg/m^2^ (HR=2.670, *P*=0.020), BMI over 30 kg/m^2^ (HR=2.160, *P*=0.038), postmenopause status (HR=2.240, *P*=0.004), the primary therapy outcome of CR (HR=4.350, *P*=0.001), residual tumor R0 (HR=2.400, *P*=0.025), or tumor invasion over 50% of the muscular layer (HR=2.800, *P*=0.004). Moreover, as shown in **Figure 6C**, the upregulation of RPP40 was also a risk factor for PFI in patients with clinical stage III–IV (HR=2.320, *P*=0.001), histological grade G3 (HR=1.650, *P*=0.017), a histological type of endometrioid (HR=1.640, *P*=0.033), age below 60 years old (HR=1.900, *P*=0.027), a BMI over 30 kg/m^2^ (HR=2.130, *P*=0.003), postmenopause status (HR=1.760, *P*=0.003), the primary therapy outcome of CR (HR=1.940, *P*=0.005), residual tumor R0 (HR=1.710, *P*=0.027), tumor invasion less than 50% of the muscular layer (HR=2.560, *P*=0.004), or tumor invasion over 50% of the muscular layer (HR=1.920, *P*=0.010).

**Figure 7:**
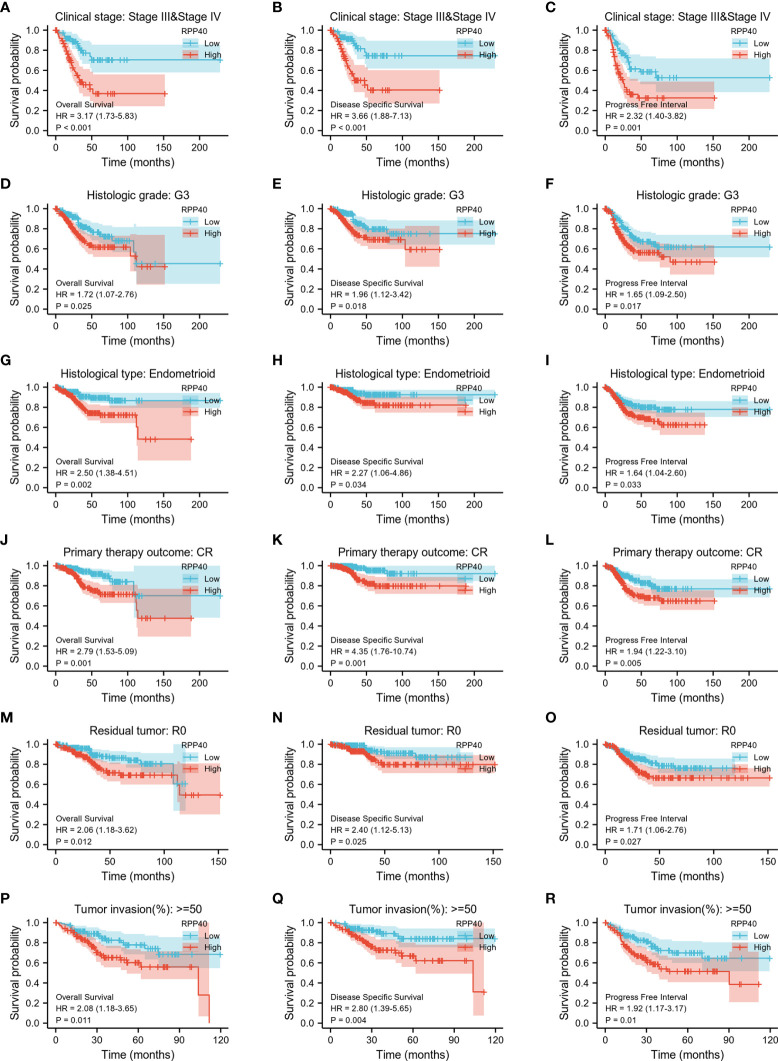
The association of clinical outcomes with RPP40 expression in UCEC patient form different subgroups based on clinicopathological factors. The result of K-M analysis showing distinct clinical outcomes of OS**(A, D, G, J, M, P)**, DSS **(B, E, H, K, N, Q)**, and PFI **(C, F, I, L, O, R)** between high- and low-RPP40-expression groups of UCEC patients in several subgroups, including clinical stage III–IV **(A-C)**, histological grade G3 **(D-F)**, the histological type of endometrioid **(G-I)**, the primary therapy outcome of CR **(J-L)**, residual tumor R0 **(M-O)**, and tumor invasion more than 50% of the muscular layer **(P-R)**.

Next, K-M analyses for the OS, DSS, and PFI of clinicopathological subgroups were performed to compare clinical outcomes between high- and low-RPP40 groups. As shown in **Figure 7** and **Supplementary Figure 3**, except DSS for tumor invasion less than 50% of the muscular layer subgroup, the RPP40 expression level exhibited a significantly prognostic value in different clinicopathological subgroups, including clinical stage III–IV, histological grade G3, the histological type of endometrioid, the primary therapy outcome of CR, residual tumor R0, tumor invasion over 50% of the muscular layer, tumor invasion less than 50% of the muscular layer, age below 60 years old, a BMI less than 30 kg/m^2^, and postmenopause status. These results indicated the prognostic value of RPP40 in UCEC was independent of the above clinicopathological factors, and the patients with low RPP40 expression possess significantly better clinical outcomes than those with high RPP40 expression.

### Identification and functional annotation of RPP40-associated DEGs in uterine corpus endometrial carcinoma

**Figure 8:**
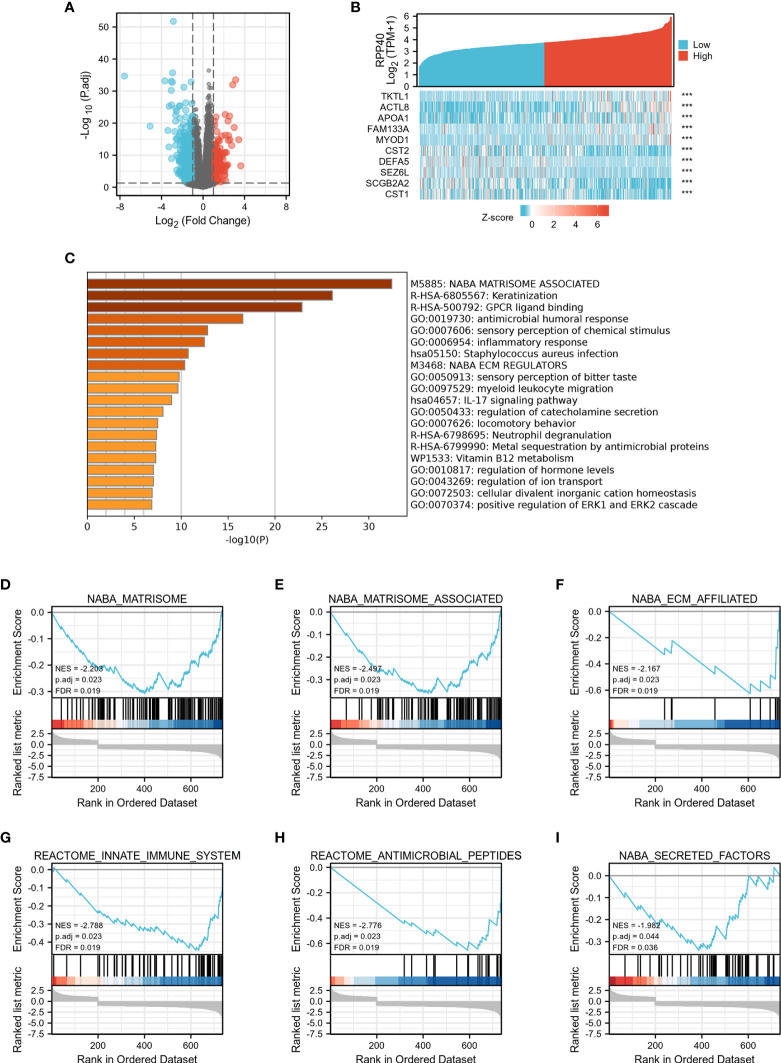
Identification and functional enrichment analysis of DEGs in UCEC patients with distinct RPP40 mRNA levels. The mRNAs of DEGs between two groups are presented by volcano plots **(A)**, and represented DEGs are shown as heatmaps **(B)**. Statistically enriched terms identified by the Metascape database are shown; the threshold of kappa score is set as 0.3; ***P < 0.001 **(C)**. Gene set enrichment analysis (GSEA) of differentially expressed mRNAs between high- and low-RPP40-expression groups in UCEC tumors has been conducted, and representative clusters are shown **(D-I)**.

In order to explore the function of RPP40 in UCEC, the DEGs between high- and low- RPP40 expression groups were identified. As shown in **Figure 8** and **Supplementary Figure 4**, 748 mRNAs (including 200 upregulated and 548 downregulated mRNAs, **Figure 8A** and **Supplementary Table 2**), 90 microRNAs (miRNAs) (including 1 upregulated and 89 downregulated miRNAs, **Supplementary Figure 4A** and **Supplementary Table 3**), and 1,408 lncRNAs (including 131 upregulated and 1,277 downregulated lncRNAs, **Supplementary Figure 4B, Supplementary Table 4**) were screened out as DEGs in the high-RPP40 group. Representative DEGs were presented in heatmaps (**Figure 8B**).

To uncover the function of RPP40 in UCEC, functional enrichment analyses of DEGs were conducted. Firstly, as shown in **Figure 8C** and **Supplementary Table 5**, an online analysis *via* “Metascape” showed that several pathways associated with RPP40 were enriched, including “NABA_MATRISOME_ASSOCIATED”, “Keratinization”, “NABA_ECM_REGULATORS”, “antimicrobial humoral response”, “inflammatory response”, “myeloid leukocyte migration”, “IL-17 signaling pathway”, “Neutrophil degranulation”, “regulation of hormone levels”, and “positive regulation of ERK1 and ERK2 cascade”. This indicated that the function of RPP40 may be mainly related to the regulation of ECM, immune or inflammatory responses, and the ERK signaling pathway. Furthermore, as shown in **Figures 8D–I** and **Supplementary Table 6**, the result of GSEA analysis showed that RPP40-associated DEGs were mainly significantly enriched in ECM-related clusters (such as NABA_MATRISOME, NABA_MATRISOME_ASSOCIATED, NABA_ECM_AFFILIATED, and NABA_SECRETED_FACTORS), and immune system-related clusters (such as REACTOME_INNATE_IMMUNE_SYSTEM, and REACTOME_ANTIMICROBIAL_PEPTIDES). Moreover, GO and KEGG enrichment analyses (**Supplementary File 2**) also showed that the enriched biological processes, molecular functions, and pathways of RPP40 were closely related to ECM regulation and an immune or inflammatory response. These results indicated that the function of RPP40 in UCEC may be associated with the regulation of ECM and immune function.

### Association of RPP40 and immune cell infiltration in uterine corpus endometrial carcinoma tumors

**Figure 9:**
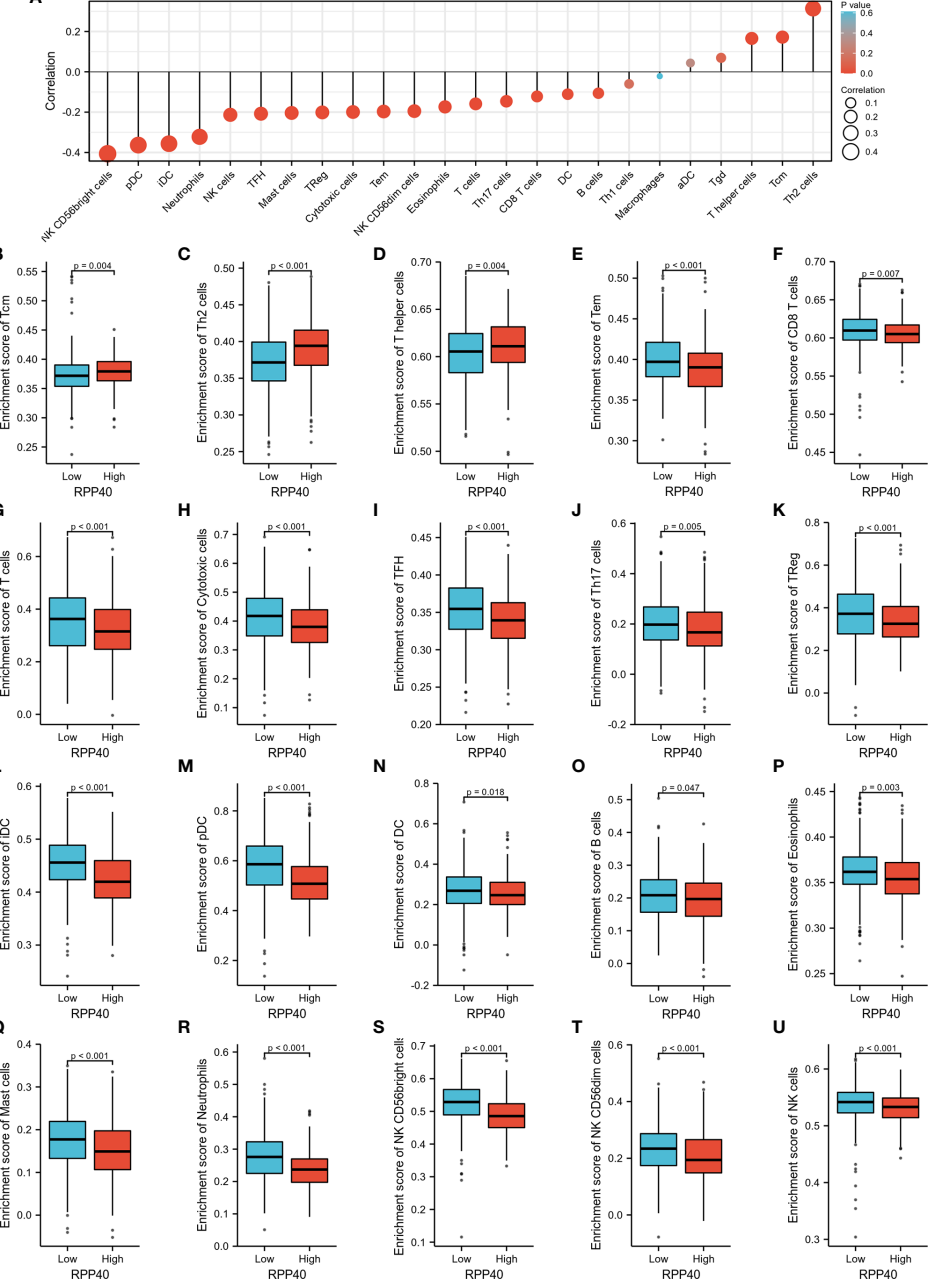
Relationships between RPP40 expression and immune cell infiltration in UCEC tumors. **(A)** The correlation of immune cell infiltration levels (24 cell types) and RPP40 mRNA expression was evaluated by Spearman’s analysis. **(B–U)** The comparison of the infiltration levels of significantly correlated immune cells, including Tcm **(B)**, Th2 cells **(C)**, T helper cells **(D)**, Tem **(E)**, CD8 T cells **(F)**, T cells **(G)**, cytotoxic cells **(H)**, TFH **(I)**, Th17 cells **(J)**, Treg **(K)**, iDC **(L)**, pDC **(M)**, DC **(N)**, B cells **(O)**, eosinophils **(P)**, mast cells **(Q)**, neutrophils **(R)**, NK CD56bright cells **(S)**, NK CD56dim cells **(T)**, and NK cells **(U)** between high- and low-RPP40-expression groups of UCEC patients.

The possible association between RPP40 and the immune system was uncovered by the functional annotation of RPP40-associated DEGs. To further confirm the possible effect of RPP40 on tumor immunity, the relationship between RPP40 expression and immune cell infiltration in UCEC was firstly determined. As shown in **Figure 9A**, the infiltration of Th2 cells (R=0.310, *P*<0.001), Tcm (R=0.145, *P*<0.001), and T helper cells (R=0.183, *P*<0.001) were significantly positively correlated with RPP40 expression. In contrast, the tumor infiltration levels of NK CD56bright cells (R=−0.365, *P*<0.001), pDC (R=−0.347, *P*<0.001), iDC (R=−0.347, *P*<0.001), neutrophils (R=−0.322, *P*<0.001), NK cells (R=−0.175, *P*<0.001), TFH (R=−0.211, *P*<0.001), mast cells (R=−0.211, *P*<0.001), Treg (R=−0.242, *P*<0.001), cytotoxic cells (R=−0.193, *P*<0.001), Tem (R=−0.213, *P*<0.001), NK CD56dim cells (R=−0.179, *P*<0.001), eosinophils (R=−0.173, *P*<0.001), T cells (R=−0.165, *P*<0.001), Th17 cells (R=−0.118, *P*<0.001), CD8 T cells (R=−0.065, *P*=0.004), DC (R=−0.139, *P*=0.009), and B cells (R=−0.125, *P*=0.013) were all significantly negatively correlated with RPP40 expression levels. Moreover, the infiltration levels of 24 immune cell types in UCEC tumor tissues between high- and low-RPP40-expression groups were compared. As shown in **Figures 9B–U**, the infiltration levels of Th2 cells, Tcm, and T helper cells were significantly increased in the high-RPP40 group. At the same time, there were 17 immune cell types (including NK CD56bright cells, pDC, iDC, neutrophils, NK cells, TFH, mast cells, Treg, cytotoxic cells, Tem, NK CD56dim cells, eosinophils, T cells, Th17 cells, CD8 T cells, DC, and B cells) significantly decreased in the high-RPP40 group. In addition, the association between the RPP40 expression and gene marker levels of immune cells in UCEC tumor tissues was evaluated *via* TIMER, as shown in **Table 4**, the RPP40 expression level in UCEC tumor tissues was closely related to the immune marker expressions of CD8^+^ T cells, T cells (general), monocytes, macrophages, neutrophils, NK cells, DC, and Th1 cells. These data indicate that RPP40 may play a specific role in the infiltration of immune cells in UCEC tumor tissues.

**Table 4:** Correlation analysis between RPP40 expression and immune cell markers in UCEC tumors.

Description	Gene markers	None		Purity	
		Cor	*P*-value	Cor	*P*-value
CD8^+^ T cells	CD8A	-0.037	0.386	-0.058	0.325
	CD8B	-0.227	***	-0.27	***
T cells (general)	CD3D	-0.158	***	-0.157	**
	CD3E	-0.167	***	-0.192	***
	CD2	-0.054	0.205	-0.056	0.338
B cells	CD19	-0.057	0.182	0.042	0.474
	CD79A	-0.112	**	-0.081	0.165
Monocyte	CD86	0.019	0.665	0.02	0.733
	CD115(CSF1R)	-0.21	***	-0.144	*
TAM	CCL2	0.04	0.357	0.09	0.126
	CD68	-0.003	0.478	0.004	0.945
	IL10	-0.03	0.272	0.064	0.272
M1 macrophage	INOS(NOS2)	-0.096	*	-0.12	*
	IRF5	0.114	**	0.109	0.062
	COX2(PTGS2)	-0.123	**	-0.058	0.325
M2 macrophage	CD163	0.132	**	0.193	***
	VSIG4	0.005	0.899	0.092	0.114
	MS4A4A	0.065	0.129	0.111	0.058
Neutrophils	CD66b(CEACAM8)	-0.188	***	-0.154	**
	CD11b(ITGAM)	-0.201	***	-0.146	**
	CCR7	-0.169	***	-0.16	**
NK cells	KIR2DL1	-0.061	0.157	-0.073	0.21
	KIR2DL3	-0.085	*	-0.131	*
	KIR2DL4	-0.03	0.491	-0.043	0.465
	KIR3DL1	-0.112	**	-0.213	***
	KIR3DL2	-0.018	0.679	-0.095	0.105
	KIR3DL3	-0.055	0.202	-0.111	0.057
	KIR2DS4	-0.108	*	-0.183	**
DC	HLA-DPB1	-0.221	***	-0.206	***
	HLA-DQB1	-0.201	***	-0.209	***
	HLA-DRA	-0.137	**	-0.129	*
	HLA-DPA1	-0.126	**	-0.111	0.057
	BDCA-1(CD1C)	-0.265	***	-0.229	***
	BDCA-4(NRP1)	-0.041	0.337	0.018	0.757
	CD11c(ITGAX)	-0.184	***	-0.164	**
Th1 cells	T-bet (TBX21)	-0.054	0.209	-0.051	0.384
	STAT4	-0.092	*	-0.064	0.271
	STAT1	0.374	***	0.367	***
	IFN-γ(IFNG)	0.025	0.554	0.014	0.814
	TNF-α(TNF)	0.085	*	0.138	*
Th2 cells	GATA3	-0.093	*	0.006	0.918
	STAT6	-0.081	0.06	0.056	0.342
	STAT5A	-0.05	0.247	0.004	0.948
	IL13	-0.039	0.369	-0.009	0.881
TFH	BCL6	-0.047	0.269	-0.049	0.404
	IL21	-0.04	0.349	-0.045	0.444
Th17 cells	STAT3	0.043	0.317	0.107	0.067
	IL17A	0.015	0.718	-0.018	0.754

*P < 0.05, **P < 0.01, ***P < 0.001. Cor: Spearman’s rho value; None: no adjusted correlation; Purity: correlation adjusted by tumor purity. The results were based on TIMER database analysis.

## Discussion

Although patients with early clinical stages of UCEC have a relatively good prognosis, the patients with advanced or relapsed UCEC still respond poorly to conventional therapies (1, 2, 4). Therefore, the mining of novel prognostic biomarkers and therapeutic targets to improve the survival rate of UCEC patients is of great scientific interest and clinical importance.

At present, the molecular function of RPP40 remains unclear since there are few studies on it. As a component of RNase P or MRP, RPP25 has been reported to promote the proliferation, migration, invasion, and cell cycle programs of cervical cancer cells (14). Furthermore, both RPP25 and RPP30, another component of RNase P and MRP, were reported as reliable prognostic risk factors for glioblastoma multiforme (11, 13). Similarly, RPP40 was also regarded as one of the promoting factors for the chemoresistance of acute myeloid leukemia (15), and the member of a prognostic signature includes seven mRNAs and could accurately predict the recurrence risks of early-stage triple-negative breast cancer (16). In addition, the result of bioinformatics analysis in this study showed that RPP40 was one of the potential prognostic genes for UCEC (**Supplementary File 1**). Therefore, we speculated that RPP40 might be involved in the tumorigenesis or progression of UCEC.

In the present study, we found that the mRNA expression of RPP40 was significantly upregulated in the tumor tissues of various cancer types, especially in UCEC. Furthermore, the protein expression of RPP40 is also significantly upregulated in UCEC tumor tissues. In addition, RPP40 expression was positively correlated with the clinical stage, histological grade, histological type, and primary therapy outcome. Based on these observations, we speculate that RPP40 might be a potential biomarker and therapeutic target of UCEC. To verify this hypothesis, we evaluated the predictive values of RPP40 in the diagnosis and prognosis of UCEC patients; the results showed that RPP40 was an effective predictor for the diagnosis of UCEC with an AUC of 0.775, a sensitivity of 0.829, and a specificity of 0.719. Furthermore, RPP40 also possessed a significant prognostic value independent of clinicopathological factors in UCEC patients, and the patients with low RPP40 expression possess significantly better clinical outcomes than those with high RPP40 expression. Therefore, we considered RPP40 as a promising prognostic biomarker for UCEC. However, studies targeting the function of RPP40 in malignant tumors are rarely reported.

The TME, composed of multiple cellular and molecular factors, has been widely implicated in tumorigenesis, progression, metastasis, and therapeutic resistance (5–7). Various components of TME, such as cancer-associated fibroblasts (CAFs), immune cells, extracellular matrix (ECM), cytokines, chemokines, and other soluble factors, act together to influence antitumor immunity, therapeutic response, and clinical outcomes (5–8). As an essential component of TME, ECM regulates cell proliferation and differentiation, and its remodeling contributes to tumor growth and metastasis (29, 30). CAFs, the main productor of ECM, interact with almost all cells within the TME that could enable them to promote the tumorigenic alterations of ECM components (29–31). Studies have confirmed that ECM stiffness and degradation always result in the proliferation, migration, and invasion of cancer cells (29). ECM stiffness was mainly regulated by integrin and transforming growth factor-β (TGF-β)–related pathways. ECM degradation was regulated primarily by matrix metalloproteinases (MMPs)/tissue inhibitors of MMP (TIMPs)-related pathways; both of these pathways have been reported to contribute to cancer cell invasion and metastasis (29, 32–34). Until now, there have been no relevant studies about the regulatory role of RPPs on ECM remodeling. In this study, “Metascape” analysis showed that several ECM-related pathways associated with RPP40 were enriched, including “NABA_MATRISOME_ASSOCIATED” and “NABA_ECM_REGULATORS”. Furthermore, the result of GSEA analysis showed that RPP40-associated DEGs were mainly significantly enriched in ECM-related clusters, such as NABA_MATRISOME, NABA_MATRISOME_ASSOCIATED, NABA_ECM_AFFILIATED, and NABA_SECRETED_FACTORS. In addition, the RPP40 expression level was significantly associated with the expression levels of ECM-related genes. In particular, RPP40 expression was positively correlated to the expressions of TGFB2, SMAD2, ITGA1, ITGB1, ITGB5, MMP1, and MMP12, and negatively correlated to the expressions of COL1A1, COL3A1, COL6A2, TGFB1, and TIMP1 (**Supplementary Figure 5**), suggesting that ECM stiffness and degradation might occur in the UCEC tumors of high-RPP40 patients. These results indicated that the function of RPP40 in UCEC might be closely related to ECM dysregulation in the TME.

Tumor-infiltrating immune cells and the cytokines, chemokines, and other soluble factors secreted by them are also crucial components of the TME (5, 7, 8). Most tumor cells express antigens that can mediate recognition by immune cells and then promote immune cell infiltration and activate the tumor immunity (35). Existing studies confirmed that tumor-infiltrating immune cells are closely associated with the clinical outcome of cancer patients (36, 37). Meanwhile, tumor cells can alter the TME and then induce immune escape and adaptive immune tolerance, which are currently considered essential for the metastases, recurrence, and therapeutic resistance of malignant tumors (5, 38). In patients with systemic autoimmune rheumatic disease, almost all RNase P and MRP complexes’ components have been reported as autoantibody targets (39–41). In addition, the expression of RPP25 was strongly correlated with immune cell infiltration levels in glioblastoma multiforme (11). Similarly, in present research, an online analysis of “Metascape” showed that several pathways associated with RPP40 were enriched, including “antimicrobial humoral response”, “inflammatory response”, “myeloid leukocyte migration”, “IL-17 signaling pathway”, “Neutrophil degranulation”, and “regulation of hormone levels”. Furthermore, the result of GSEA analysis showed that RPP40-associated DEGs were mainly significantly enriched in immune system–related clusters, such as “REACTOME_INNATE_IMMUNE_SYSTEM” and “REACTOME_ANTIMICROBIAL_PEPTIDES”. Moreover, the upregulation of RPP40 was significantly negatively correlated with the tumor infiltration levels of most of immune cell types, such as NK cells, DCs, cytotoxic cells, and CD 8 T cells. DCs are a group of specialized antigen-presenting cells; CD 8 T cells are essential cancer antigen recognition cells that act together and have critical roles in initiating and regulating anti-tumor immune responses (42, 43). NK cells and cytotoxic cells are important effectors of antitumor immunity and can directly kill cancer cells (44, 45). These results indicated that the function of RPP40 in UCEC might also be closely related to the TME.

Based on the above results, we believe that RPP40 is a promising prognostic biomarker correlated with the TME in UCEC. Meanwhile, the mechanism underlying the regulatory function of RPP40 on the TME is still not clear. We notice that RPP40-related DEGs were also significantly enriched in the “positive regulation of ERK1 and ERK2 cascade” in the functional annotation analysis based on “Metascape”. As protein-serine/threonine kinases, both ERK1 and ERK2 are essential components of the Ras-Raf-MEK-ERK signaling cascade, which has been reported to regulate cell proliferation, survival, differentiation, metabolism, adhesion, and migration (46). In malignant tumors, the ERK signaling pathway has been confirmed to promote the transformation of fibroblasts to CAFs in colorectal cancer (47). Furthermore, the ERK1/2 signaling pathway has also been reported as a promoting factor of tumor ECM degradation and angiogenesis, contributing to the proliferation, invasion, and metastasis of malignant tumors (48). Similarly, ERK1/2 signaling cascade has been proven to regulate the tumor immune microenvironment by recruiting immune cells in glioblastoma (49). Therefore, we speculate that the regulatory mechanism of RPP40 in the TME of UCEC may be closely related to the regulation of ERK signaling pathways, whereas further verification studies are needed.

Although we revealed a potential role and the possible mechanism of RPP40 in UCEC tumorigenesis and prognosis, there are still several limitations in this research. Firstly, we just evaluated the association of RPP40 expression and the expression of ECM-related genes in UCEC tumors based on the TCGA database, while CAFs are the main productor of ECM. Therefore, the association analysis between RPP40 expression in tumor cells and the ECM-related gene expressions in CAFs is more convincing. Secondly, further *in vivo* and *in vitro* experiments, and the confirming studies in protein levels are all needed to verify the effect and direct mechanism of RPP40 in UCEC.

## Conclusions

The upregulation of RPP40 might play an important role in the tumorigenesis and progression of UCEC by regulating the TME and exhibiting a reliable diagnostic and prognostic value for clinical outcomes. The results of this study indicate the possibility of RPP40 as a promising biomarker and therapeutic target for UCEC.

## Data availability statement

All data used in this research was acquired from the TCGA database. This data can be found online: https://portal.gdc.cancer.gov/. The original contributions presented in the study are included in the article/**Supplementary Material**. Further inquiries can be directed to the corresponding author.

## Author contributions

JT, XT, JM and MH: project investigation. JT and XT: methodology. JT and XT: writing–original draft. JT and LH: writing–review and editing. LH: project administration and supervision. All authors contributed to this article and approved the submitted version of the manuscript.

## Funding

This work was supported by the National Natural Science Foundation of China (No. 82001527), and Open Project of Hubei Key Laboratory from Renmin Hospital of Wuhan University (No. 2021KFY003).

## Conflict of interest

The authors declare that the research was conducted in the absence of any commercial or financial relationships that could be construed as a potential conflict of interest.

## Publisher’s note

All claims expressed in this article are solely those of the authors and do not necessarily represent those of their affiliated organizations, or those of the publisher, the editors and the reviewers. Any product that may be evaluated in this article, or claim that may be made by its manufacturer, is not guaranteed or endorsed by the publisher.
