# Supplementary material for: RPP40 is a prognostic biomarker and correlated with tumor microenvironment in uterine corpus endometrial carcinoma
Source: Front Oncol. 2022 Aug 24;12:957472. doi: 10.3389/fonc.2022.957472 (PMC9448918; doi:10.3389/fonc.2022.957472)
Supplement: Supplementary file 1 [file DataSheet_1.docx]

RPP40 is a Prognostic Biomarker and Correlated with Tumor Microenvironment in Uterine Corpus Endometrial Carcinoma

Supplementary Material

## Supplementary Figures


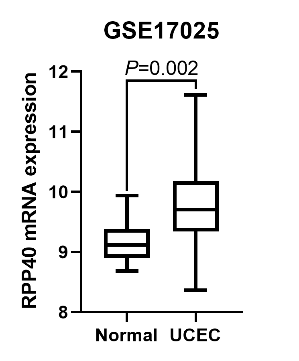


**Supplementary Figure 1.** Unpaired analysis of the mRNA expression levels of RPP40 in 91 UCEC samples and 12 normal samples from the GSE17025 dataset.

**
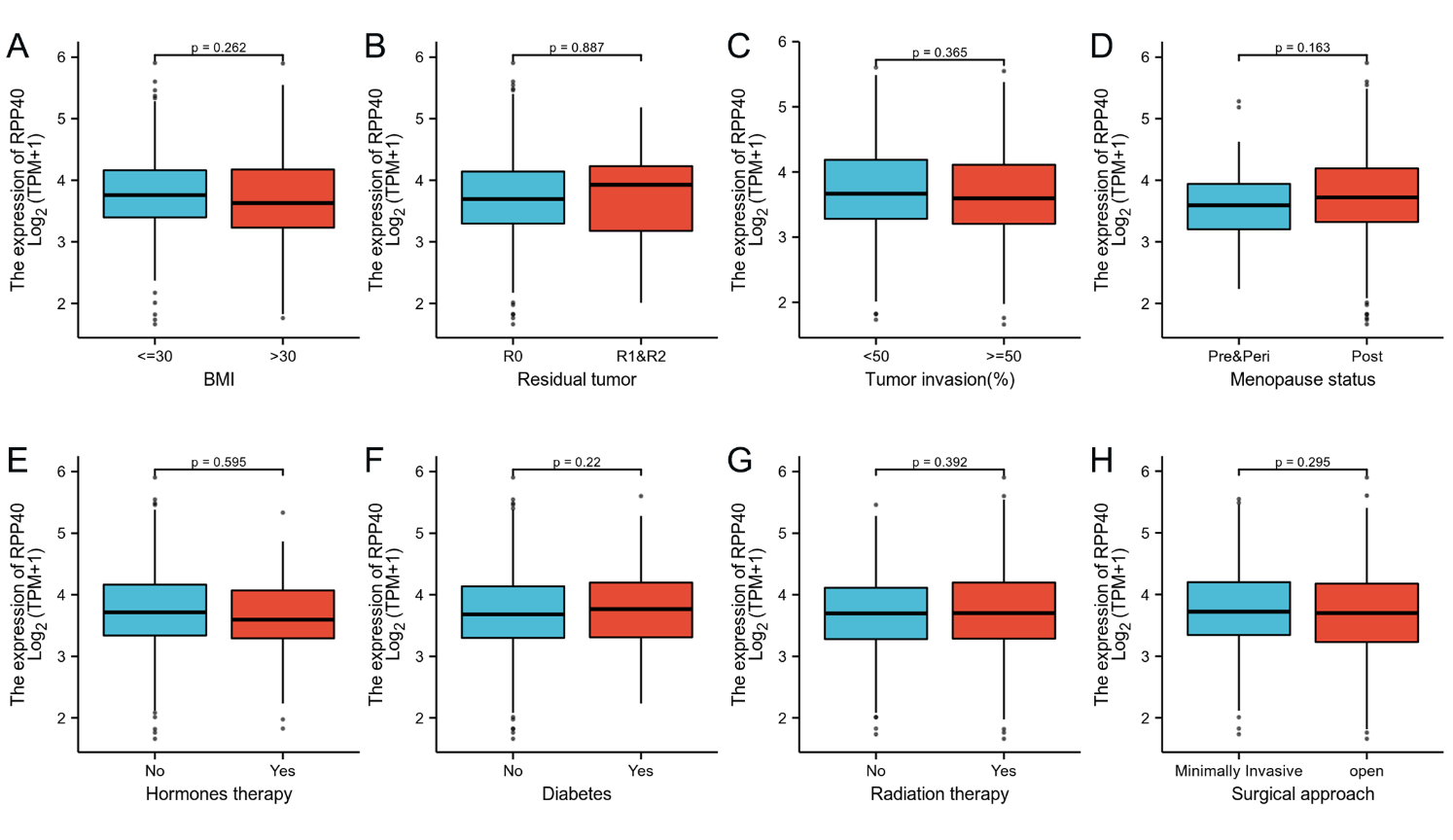
**

**Supplementary Figure 2.** The association between RPP40 expression and clinicopathological characteristics in UCEC patients. The expression differences of RPP40 between distinct subgroups of UCEC patients based on different clinicopathological characteristics, including body mass index (BMI) (A), residual tumor (B), tumor invasion (C), menopause status (D), hormones therapy (E), diabetes (F), radiation therapy (G), and surgical approach (H).


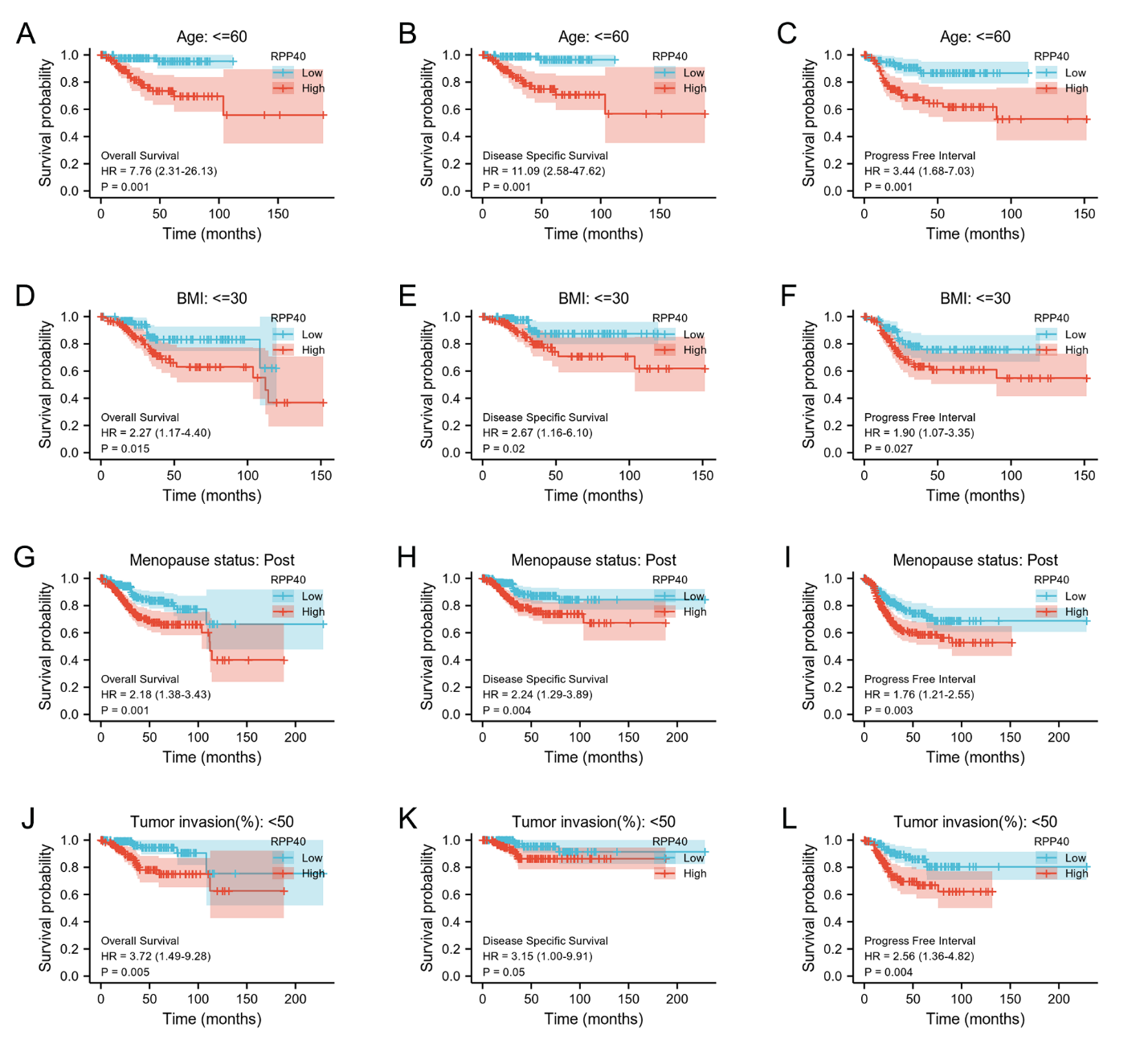


**Supplementary Figure 3.** The association of clinical outcomes with RPP40 expression in UCEC patient form different subgroups based on clinicopathological factors. The result of K-M analysis showing distinct clinical outcomes of OS (A, D, G, and J), DSS (B, E, H, and K), and PFI (C, F, I, and L) between high- and low-RPP40 expression groups of UCEC patients in several subgroups, including age below 60 years (A-C), BMI less than 30 kg/m2 (D-F), post menopause status (G-I), and tumor invasion less than 50% of muscular layer (J-L).


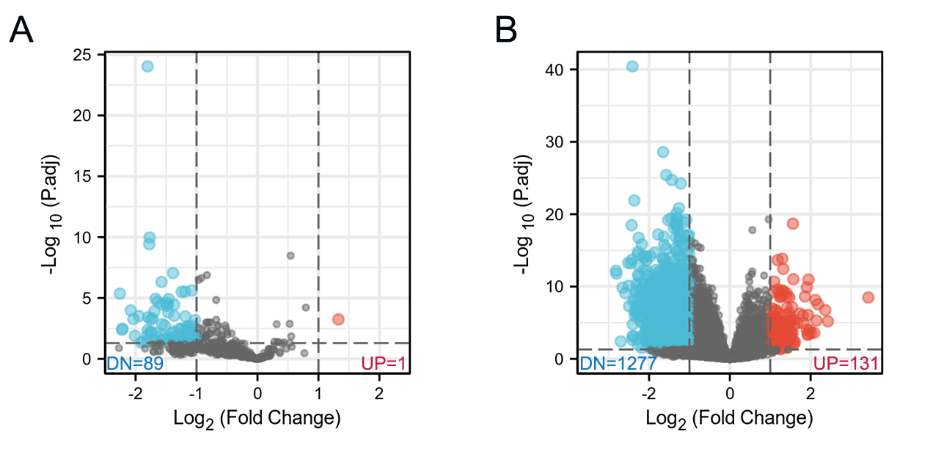


**Supplementary Figure 4.** Identification of RPP40-Associated Differentially Expressed Genes (DEGs) in UCEC. Differentially Expressed miRNAs (A) and lncRNAs (B) between high- and low-RPP40 expression groups of UCEC patients are presented by volcano plots. P<0.05 and |log2 Fold change|>1.0 were set as thresholds for DEGs with statistical significance.


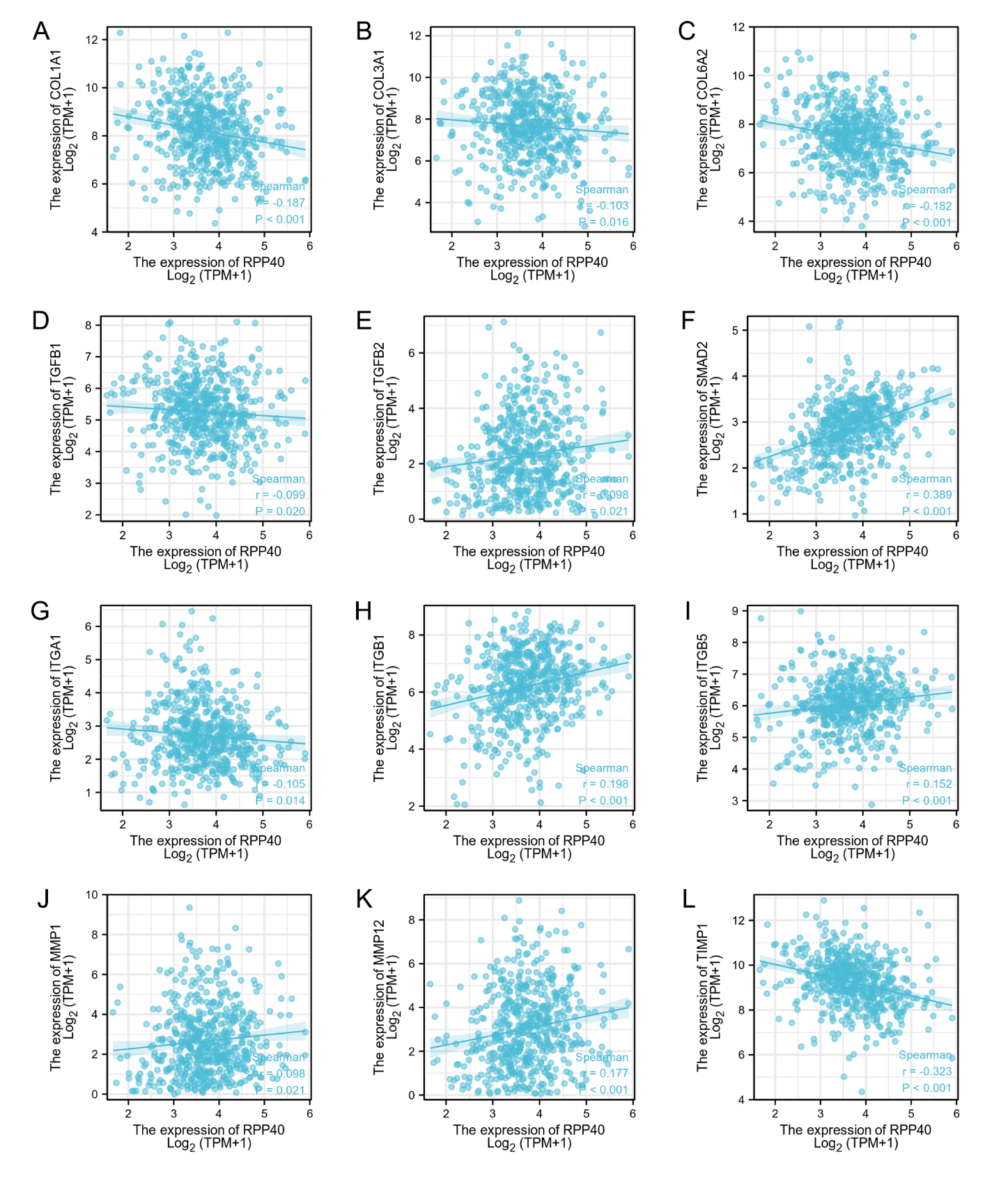


**Supplementary Figure 5.** Relationships between RPP40 expression and the expression of ECM-related genes in UCEC tumors. Based on TCGA database, spearman’s analysis was used to evaluated the association between RPP40 expression and the expression of ECM-related genes, including COL1A1 (A), COL3A1 (B), COL6A2 (C), TGFB1 (D), TGFB2 (E), SMAD2 (F), ITGA1 (G), ITGB1 (H), ITGB5 (I), MMP1 (J), MMP12 (K), and TIMP1 (L).

## Supplementary Tables

**Supplementary Table 1. Sample size of each subgroup.**

| **Characteristic** | **Low-RPP40**  **expression (n)** | **High-RPP40**  **expression (n)** |
| --- | --- | --- |
| Clinical stage | 276 | 276 |
| Stage I-II | 210 | 183 |
| Stage III-IV | 66 | 93 |
| Primary therapy outcome | 249 | 231 |
| CR | 238 | 204 |
| PR & PD & SD | 11 | 27 |
| Age | 274 | 275 |
| <=60 | 113 | 93 |
| >60 | 161 | 182 |
| BMI, | 272 | 257 |
| <=30 | 99 | 113 |
| >30 | 163 | 144 |
| Histological type | 276 | 276 |
| Endometrioid | 234 | 176 |
| Mixed & Serous | 42 | 100 |
| Residual tumor | 211 | 202 |
| R0 | 193 | 182 |
| R1-2 | 18 | 20 |
| Histologic grade | 272 | 269 |
| G1-2 | 147 | 71 |
| G3 | 125 | 198 |
| Tumor invasion (%) | 250 | 224 |
| <50 | 134 | 125 |
| >=50 | 116 | 99 |
| Menopause status | 253 | 253 |
| Pre & Peri | 30 | 22 |
| Post | 223 | 231 |
| Hormones therapy | 175 | 169 |
| No | 148 | 149 |
| Yes | 27 | 20 |
| Diabetes | 229 | 222 |
| No | 171 | 157 |
| Yes | 58 | 65 |
| Radiation therapy | 267 | 260 |
| No | 142 | 137 |
| Yes | 125 | 123 |
| Surgical approach | 266 | 264 |
| Minimally Invasive | 103 | 105 |
| open | 163 | 159 |
| OS event | 276 | 276 |
| Alive | 246 | 212 |
| Dead | 30 | 64 |
| DSS event | 276 | 274 |
| Alive | 256 | 231 |
| Dead | 20 | 43 |
| PFI event | 276 | 276 |
| Alive | 226 | 197 |
| Dead | 50 | 79 |

All above information of UCEC patients were acquired from TCGA database.

**Supplementary Table 2.** Top 20 upregulated and downregulated mRNAs in high-RPP40 group compared with low-RPP40 group of UCEC patients.

| **Gene-UP** | **log2FC** | ***P* value** | ***P* adj** | **Gene-DN** | **log2FC** | ***P* value** | ***P* adj** |
| --- | --- | --- | --- | --- | --- | --- | --- |
| GAGE2A | 3.63 | 1.76E-08 | 2.06E-07 | DEFA5 | -7.56 | 3.17E-39 | 2.06E-35 |
| CT45A1 | 3.44 | 1.52E-17 | 1.54E-15 | DEFA6 | -5.10 | 3.24E-22 | 9.02E-20 |
| TKTL1 | 3.13 | 5.66E-38 | 3.23E-34 | CACNA1S | -3.67 | 1.33E-37 | 6.75E-34 |
| MYOD1 | 3.00 | 9.34E-22 | 2.31E-19 | GP2 | -3.39 | 1.01E-20 | 1.97E-18 |
| ACTL8 | 2.86 | 3.4E-36 | 1.11E-32 | REG3A | -3.29 | 4.65E-15 | 2.66E-13 |
| PAGE2 | 2.76 | 1.35E-15 | 8.77E-14 | CALML3 | -3.26 | 5.19E-29 | 5.63E-26 |
| CALCA | 2.59 | 1.35E-17 | 1.4E-15 | SCGB2A2 | -3.07 | 2.39E-34 | 6.05E-31 |
| CALCB | 2.55 | 1.13E-25 | 6.72E-23 | SEZ6L | -3.06 | 1.85E-37 | 8.44E-34 |
| FOLR3 | 2.45 | 5.22E-19 | 7.37E-17 | CST1 | -3.04 | 1.51E-29 | 1.97E-26 |
| NTS | 2.43 | 2.67E-17 | 2.53E-15 | LY6L | -3.03 | 5.66E-11 | 1.25E-09 |
| FAM133A | 2.41 | 5.34E-24 | 2.21E-21 | MUC5AC | -2.95 | 5E-37 | 1.9E-33 |
| PDYN | 2.38 | 6.77E-09 | 8.79E-08 | CST2 | -2.93 | 2.66E-40 | 2.02E-36 |
| KLHL1 | 2.27 | 1.31E-08 | 1.58E-07 | MYH7B | -2.86 | 7.37E-57 | 1.68E-52 |
| CTCFL | 2.25 | 3.89E-13 | 1.45E-11 | KRT31 | -2.72 | 2.35E-20 | 4.26E-18 |
| MAGEA10 | 2.22 | 3.12E-08 | 3.43E-07 | BPIFA1 | -2.65 | 1.26E-15 | 8.22E-14 |
| ZFP42 | 2.20 | 1.86E-08 | 2.17E-07 | LY6G6C | -2.64 | 4.72E-26 | 3.08E-23 |
| FOXR2 | 2.18 | 0.003224 | 0.009082 | C6orf15 | -2.64 | 5.7E-24 | 2.32E-21 |
| SOX11 | 2.15 | 4.07E-19 | 5.8E-17 | MUC6 | -2.62 | 3.21E-28 | 2.81E-25 |
| APOA1 | 2.14 | 2.92E-26 | 1.99E-23 | BPIFB2 | -2.59 | 6.62E-18 | 7.38E-16 |
| MAGEA4 | 2.11 | 6.15E-07 | 4.76E-06 | MUC5B | -2.56 | 2.59E-27 | 1.88E-24 |

Gene-UP: upregulated mRNAs; Gene-DN: downregulated mRNAs; log2FC: log2Fold Change; *P* adj: adjusted *P*-value

**Supplementary Table 3.** The upregulated and top 20 downregulated mRNAs in high-RPP40 group compared with low-RPP40 group of UCEC patients.

| **Gene-UP** | **log2FC** | ***P* value** | ***P* adj** | **Gene-DN** | **log2FC** | ***P* value** | ***P* adj** |
| --- | --- | --- | --- | --- | --- | --- | --- |
| MIR4477B | 1.33 | 0.0001356 | 0.0005666 | MIR581 | -2.26 | 5.531E-07 | 4.333E-06 |
|  |  |  |  | MIR181A2 | -2.22 | 0.0012756 | 0.0040454 |
|  |  |  |  | MIR938 | -2.21 | 0.0010023 | 0.0032738 |
|  |  |  |  | MIR4802 | -2.08 | 1.976E-05 | 0.0001036 |
|  |  |  |  | MIR1296 | -2.04 | 0.0001227 | 0.0005185 |
|  |  |  |  | MIR640 | -2.01 | 0.0047944 | 0.0127951 |
|  |  |  |  | MIR1256 | -1.94 | 6.768E-05 | 0.0003075 |
|  |  |  |  | MIR151A | -1.91 | 0.0207983 | 0.0450334 |
|  |  |  |  | MIR4652 | -1.87 | 0.0166057 | 0.0372333 |
|  |  |  |  | MIR5692C2 | -1.81 | 0.0043089 | 0.0116779 |
|  |  |  |  | MIR4737 | -1.80 | 1.224E-27 | 9.465E-25 |
|  |  |  |  | MIR3128 | -1.80 | 0.0167271 | 0.0374686 |
|  |  |  |  | MIR4714 | -1.80 | 0.0013744 | 0.0043217 |
|  |  |  |  | MIR548K | -1.79 | 8.443E-05 | 0.0003731 |
|  |  |  |  | MIR604 | -1.78 | 1.493E-11 | 3.812E-10 |
|  |  |  |  | MIR3609 | -1.77 | 3.601E-12 | 1.059E-10 |
|  |  |  |  | MIR624 | -1.76 | 0.0019749 | 0.0059382 |
|  |  |  |  | MIR548V | -1.75 | 0.0001387 | 0.0005783 |
|  |  |  |  | MIR2278 | -1.74 | 0.0022645 | 0.0066839 |
|  |  |  |  | MIR4755 | -1.72 | 0.000111 | 0.0004745 |

Gene-UP: upregulated miRNAs; Gene-DN: downregulated miRNAs; log2FC: log2Fold Change; *P* adj: adjusted *P*-value

**Supplementary Table 4.** Top 20 upregulated and downregulated lncRNAs in high-RPP40 group compared with low-RPP40 group of UCEC patients.

| **Gene-UP** | **log2FC** | ***P* value** | ***P* adj** | **Gene-DN** | **log2FC** | ***P* value** | ***P* adj** |
| --- | --- | --- | --- | --- | --- | --- | --- |
| SOX1-OT | 3.43 | 1.66E-10 | 3.27E-09 | DOCK4-AS1 | -2.82 | 1.3E-14 | 6.73E-13 |
| LINC01639 | 2.43 | 8.68E-07 | 6.46E-06 | AL034349.1 | -2.81 | 3.4E-14 | 1.62E-12 |
| ESRG | 2.36 | 1.52E-08 | 1.8E-07 | AC096733.1 | -2.70 | 0.001228 | 0.003916 |
| U95743.1 | 2.18 | 1.84E-09 | 2.78E-08 | AF178030.1 | -2.66 | 5.01E-12 | 1.42E-10 |
| AC074389.2 | 2.14 | 6.48E-07 | 4.99E-06 | RASA2-IT1 | -2.59 | 3.54E-11 | 8.24E-10 |
| AL358394.1 | 2.12 | 3.93E-10 | 7.07E-09 | PTPRJ-AS1 | -2.52 | 8.04E-16 | 5.44E-14 |
| MMEL1-AS1 | 2.09 | 5.29E-05 | 0.000247 | AL353771.1 | -2.51 | 2.42E-12 | 7.46E-11 |
| MAGEA4-AS1 | 2.01 | 5.56E-05 | 0.000258 | AC073651.1 | -2.49 | 3.64E-09 | 5.07E-08 |
| FLJ36000 | 1.97 | 2.26E-05 | 0.000116 | PACRG-AS3 | -2.45 | 9E-16 | 6.01E-14 |
| LINC01976 | 1.95 | 2.84E-13 | 1.08E-11 | AL008636.1 | -2.43 | 1.43E-21 | 3.33E-19 |
| AC090809.1 | 1.94 | 8.62E-05 | 0.00038 | AC016027.3 | -2.42 | 2.54E-16 | 1.91E-14 |
| MIR663AHG | 1.93 | 8.72E-08 | 8.5E-07 | AC016705.1 | -2.41 | 2.48E-45 | 3.77E-41 |
| AL021395.1 | 1.92 | 0.000114 | 0.000487 | PLCB1-IT1 | -2.41 | 0.0007 | 0.002392 |
| FOXD3-AS1 | 1.91 | 4.35E-12 | 1.25E-10 | BACH1-IT3 | -2.41 | 0.000178 | 0.000719 |
| AC021088.1 | 1.90 | 1.64E-05 | 8.76E-05 | AL035634.1 | -2.40 | 2.64E-06 | 1.73E-05 |
| LINC02575 | 1.86 | 1.17E-10 | 2.4E-09 | ZBTB20-AS5 | -2.39 | 5.64E-10 | 9.75E-09 |
| AC107057.1 | 1.82 | 1.72E-06 | 1.19E-05 | LPP-AS1 | -2.39 | 0.00019 | 0.000762 |
| PDE11A-AS1 | 1.81 | 1.06E-07 | 1.01E-06 | AL355075.4 | -2.37 | 2.47E-25 | 1.26E-22 |
| GPR1-AS | 1.73 | 5.25E-08 | 5.39E-07 | AL359095.1 | -2.36 | 2.41E-08 | 2.72E-07 |
| AC004888.1 | 1.66 | 1.62E-06 | 1.13E-05 | AC012568.1 | -2.35 | 1.32E-07 | 1.23E-06 |

Gene-UP: upregulated lncRNAs; Gene-DN: downregulated lncRNAs; log2FC: log2Fold Change; *P* adj: adjusted *P*-value

**Supplementary Table 5.** Enrichment analysis of differentially expressed mRNAs by Metascape database.

| **GO** | **Category** | **Description** | **Count** | **Log10(P)** | **Log10(q)** |
| --- | --- | --- | --- | --- | --- |
| M5885 | Canonical Pathways | NABA MATRISOME ASSOCIATED | 86 | -32.45 | -28.1 |
| R-HSA-6805567 | Reactome Gene Sets | Keratinization | 43 | -26.1 | -22.05 |
| R-HSA-500792 | Reactome Gene Sets | GPCR ligand binding | 57 | -22.87 | -19 |
| GO:0019730 | GO Biological Processes | antimicrobial humoral response | 25 | -16.57 | -13 |
| GO:0007606 | GO Biological Processes | sensory perception of chemical stimulus | 46 | -12.81 | -9.64 |
| GO:0006954 | GO Biological Processes | inflammatory response | 45 | -12.47 | -9.33 |
| hsa05150 | KEGG Pathway | Staphylococcus aureus infection | 18 | -10.75 | -7.72 |
| M3468 | Canonical Pathways | NABA ECM REGULATORS | 27 | -10.38 | -7.37 |
| GO:0050913 | GO Biological Processes | sensory perception of bitter taste | 12 | -9.77 | -6.82 |
| GO:0097529 | GO Biological Processes | myeloid leukocyte migration | 19 | -9.66 | -6.72 |
| hsa04657 | KEGG Pathway | IL-17 signaling pathway | 16 | -8.98 | -6.2 |
| GO:0050433 | GO Biological Processes | regulation of catecholamine secretion | 12 | -8.07 | -5.4 |
| GO:0007626 | GO Biological Processes | locomotory behavior | 20 | -7.5 | -4.88 |
| R-HSA-6798695 | Reactome Gene Sets | Neutrophil degranulation | 34 | -7.38 | -4.8 |
| R-HSA-6799990 | Reactome Gene Sets | Metal sequestration by antimicrobial proteins | 5 | -7.3 | -4.74 |
| WP1533 | WikiPathways | Vitamin B12 metabolism | 11 | -7.29 | -4.74 |
| GO:0010817 | GO Biological Processes | regulation of hormone levels | 34 | -7.04 | -4.52 |
| GO:0043269 | GO Biological Processes | regulation of ion transport | 42 | -7.04 | -4.52 |
| GO:0072503 | GO Biological Processes | cellular divalent inorganic cation homeostasis | 21 | -6.91 | -4.4 |
| GO:0070374 | GO Biological Processes | positive regulation of ERK1 and ERK2 cascade | 21 | -6.87 | -4.38 |

**Supplementary Table 6.** Gene Set Enrichment Analysis (GSEA) of differentially expressed mRNAs between high- and low-RPP40 expression groups in UCEC tumors.

| **Description** | **enrichmentScore** | **NES** | ***P*-value** | ***P*-adjust** |
| --- | --- | --- | --- | --- |
| NABA_MATRISOME | -0.3078 | -2.2028 | 0.0013 | 0.0232 |
| NABA_MATRISOME_ASSOCIATED | -0.3619 | -2.4969 | 0.0013 | 0.0232 |
| REACTOME_INNATE_IMMUNE_SYSTEM | -0.4452 | -2.7880 | 0.0013 | 0.0232 |
| REACTOME_POST_TRANSLATIONAL_PROTEIN_MODIFICATION | -0.3899 | -2.2701 | 0.0014 | 0.0232 |
| REACTOME_ANTIMICROBIAL_PEPTIDES | -0.6546 | -2.7756 | 0.0015 | 0.0232 |
| NABA_ECM_AFFILIATED | -0.6265 | -2.1669 | 0.0016 | 0.0232 |
| REACTOME_KERATINIZATION | -0.3769 | -2.1375 | 0.0028 | 0.0340 |
| NABA_SECRETED_FACTORS | -0.3401 | -1.9821 | 0.0041 | 0.0445 |
| NABA_ECM_REGULATORS | -0.4010 | -1.8984 | 0.0058 | 0.0556 |
| REACTOME_DISEASES_OF_METABOLISM | -0.5457 | -1.8877 | 0.0065 | 0.0556 |
| KEGG_PATHWAYS_IN_CANCER | -0.5504 | -1.8208 | 0.0084 | 0.0586 |
| WP_ESC_PLURIPOTENCY_PATHWAYS | -0.5506 | -1.8216 | 0.0084 | 0.0586 |
| REACTOME_DEVELOPMENTAL_BIOLOGY | -0.2857 | -1.8529 | 0.0092 | 0.0586 |
| REACTOME_POST_TRANSLATIONAL_MODIFICATION_SYNTHESIS_OF_GPI_ANCHORED_PROTEINS | -0.5064 | -1.7966 | 0.0095 | 0.0586 |
| KEGG_OLFACTORY_TRANSDUCTION | -0.3789 | -1.7940 | 0.0116 | 0.0668 |
| REACTOME_OLFACTORY_SIGNALING_PATHWAY | -0.3739 | -1.7122 | 0.0225 | 0.1211 |
| REACTOME_FORMATION_OF_THE_CORNIFIED_ENVELOPE | -0.3405 | -1.5593 | 0.0405 | 0.2044 |
| WP_MAPK_SIGNALING_PATHWAY | -0.4401 | -1.6056 | 0.0428 | 0.2044 |
| KEGG_MAPK_SIGNALING_PATHWAY | -0.4092 | -1.5716 | 0.0472 | 0.2135 |
| WP_FOCAL_ADHESIONPI3KAKTMTORSIGNALING_PATHWAY | -0.4138 | -1.5096 | 0.0697 | 0.2998 |
